# Etiology and longitudinal kidney outcomes in children with nephrocalcinosis: a retrospective cohort study

**DOI:** 10.3389/fped.2026.1779744

**Published:** 2026-03-06

**Authors:** Mihriban İnözü, Sibel Çetince Şenses, Özlem Yüksel Aksoy, Burcu Yazıcıoğlu, Sare Gülfem Özlü, Fatma Şemsa Çaycı, Umut Selda Bayrakçı

**Affiliations:** 1 Faculty of Medicine, Department of Pediatric Nephrology, Ankara Yıldırım Beyazıt University, Ankara, Türkiye; 2 Ankara Bilkent City Hospital, Department of Pediatric Nephrology, Ankara, Türkiye; 3 Faculty of Medicine, Department of Pediatric Nephrology, Gazi University, Ankara, Türkiye; 4 Faculty of Medicine, University of Health Sciences, Department of Pediatric Nephrology, Ankara, Türkiye

**Keywords:** children, etiology, kidney function, long-term outcome, nephrocalcinosis

## Abstract

**Background:**

Nephrocalcinosis refers to the deposition of calcium salts within the kidney parenchyma and is a condition encountered in various metabolic, genetic, and systemic disorders in childhood. Nephrocalcinosis is increasingly recognized in children; however, data on long-term kidney outcomes and prognostic factors remain limited.

**Methods:**

This study was designed as a retrospective cohort study. We evaluated the clinical characteristics, etiological spectrum, and longitudinal kidney outcomes of children with nephrocalcinosis. Changes in estimated glomerular filtration rate (eGFR) over follow-up were assessed, and factors associated with kidney function decline were analyzed using multivariable regression.

**Results:**

Among 73 children with nephrocalcinosis, 43 were included in longitudinal analyses with a median follow-up of 52 months. Median eGFR declined significantly over time (*p* = 0.006). Kidney outcomes varied according to underlying etiology, with lower final eGFR observed in children with hereditary or genetic causes. In multivariable analysis, systemic/syndromic etiology, history of urinary tract infection, and older age at diagnosis were independently associated with greater eGFR decline, whereas traditional metabolic risk factors were not independently associated with longitudinal changes in kidney function.

**Conclusion:**

Our findings suggest that pediatric nephrocalcinosis may be associated with a decline in kidney function over long-term follow-up and that kidney outcomes are influenced by underlying etiological and clinical factors. Given the paucity of studies addressing the long-term prognosis of nephrocalcinosis, these results highlight the need for early diagnosis, careful etiological evaluation, individualized risk stratification, and close follow-up, particularly in high-risk subgroups. Furthermore, our study underscores the need for prospective studies to better characterize prognostic factors and optimize long-term care in children with nephrocalcinosis.

## Introduction

Nephrocalcinosis is defined as the deposition of calcium phosphate or calcium oxalate salts within the kidney parenchyma, including the tubular epithelium and interstitial kidney tissue (1, 2). Although nephrocalcinosis may occur at the molecular, microscopic, or macroscopic level, in routine clinical practice the term most commonly refers to macroscopic kidney calcifications that are detectable by imaging studies (3). The routine use of ultrasound has led to increased detection of nephrocalcinosis in both children and adults (4). In most patients, nephrocalcinosis predominantly involves the kidney medulla and appears on ultrasonography as bilaterally symmetric areas of increased echogenicity within the kidney pyramids (3).

Although the exact pathogenesis of nephrocalcinosis remains incompletely understood, medullary nephrocalcinosis is widely considered to be a consequence of hypercalciuria. An increased urinary calcium load may result either from enhanced intestinal calcium absorption due to extrarenal causes or from impaired tubular calcium reabsorption within the kidney (5).

Because nephrocalcinosis develops within the medullary interstitium, it may induce relative hypoxia and inflammation. Nephrocalcinosis may be associated with progressive kidney dysfunction, particularly in a limited number of conditions such as primary hyperoxaluria, familial hypomagnesemia with hypercalciuria and nephrocalcinosis (FHHNC), Dent disease, and Lowe syndrome. However, the mechanisms underlying the association between nephrocalcinosis and progressive kidney dysfunction in only certain conditions are not yet fully understood (5).

A wide range of metabolic and genetic factors, including both inherited and acquired disorders, may lead to nephrocalcinosis (1). A personalized, patient-centered approach is essential in the initial diagnostic evaluation of individuals affected by nephrocalcinosis. An early age at presentation together with a positive family history or consanguinity should raise suspicion for a hereditary tubulopathy (3). Accordingly, detailed metabolic evaluation, with consideration of genetic testing when appropriate, is recommended, as clarification of the underlying etiology has important implications for prognosis, management, and family counseling.

Studies evaluating long-term outcomes and factors influencing prognosis in children with nephrocalcinosis remain limited (4). This study aimed to characterize the clinical and demographic features of children with nephrocalcinosis and to identify underlying metabolic, genetic, and systemic causes. We further aimed to evaluate longitudinal changes in kidney function, with particular emphasis on factors associated with these changes.

## Materials and methods

This retrospective cohort study was conducted at a single medical center and included patients who were followed at the Pediatric Nephrology Department of Ankara Bilkent City Hospital between September 2019 and September 2025. A total of 73 children aged 1–18 years who were diagnosed with bilateral nephrocalcinosis by ultrasonography were included. Patients whose medical records were not available were excluded. The study protocol was approved by the hospital Ethics Committee (date 12 June 2024/approval, TABED 2/238/2024), and the study was conducted in accordance with the Helsinki Declaration of 1964.

Demographic and clinical data were obtained through the hospital database. Participants' sex; dates of birth, initial presentation, and last documented follow-up; antenatal history; parental consanguinity; family history; and initial symptoms were recorded. A history of urinary tract infections (UTIs), other systemic anomalies or diseases, and prior surgical interventions was also documented. Kidney stones and other urinary tract anomalies were evaluated by ultrasonography and recorded. Etiologic diagnoses were categorized into four clinically meaningful groups: (1) hereditary or genetic causes, (2) metabolic or endocrine causes, (3) systemic or syndromic causes, and (4) unknown etiology.

Urinary tract infection (UTI) was defined by the presence of typical signs and symptoms (fever, dysuria, or flank tenderness) and confirmed by positive urinalysis and urine culture (>100,000 colony-forming units of a single organism). Recurrent UTIs were defined as episodes of UTI occurring after the completion of previous UTI treatment (6).

At diagnosis, body height, laboratory findings were recorded. Laboratory assessment included measurements of serum creatinine, urea, electrolytes, uric acid, pH, bicarbonate and parathormone, along with urinalysis, urine culture, urinary amino acid analysis, and evaluation of urinary solute excretion using either 24-h urine collections or solute-to-creatinine ratios from spot urine samples in infants and toddlers. Metabolic abnormalities—hypercalciuria, hypocitraturia, hyperoxaluria, hyperuricosuria, and cystinuria—were assessed according to established pediatric reference ranges for urinary constituents (7). For patients with available follow-up data, serum creatinine levels and height measurements at the last visit were also recorded. Among patients included in the longitudinal eGFR analysis, no cases of vesicoureteral reflux or ureteropelvic stenosis were identified, and patient with an unilateral atrophic kidney was excluded from this analysis. Estimated glomerular filtration rates (eGFRs) at initial presentation and at the last visit in patients with available follow-up were calculated using the modified Schwartz formula (8). The difference between eGFR at initial presentation (eGFR_initial) and at the final follow-up visit (eGFR_final) was calculated and defined as ΔGFR. Because follow-up eGFR measurements were obtained at variable and non-standardized time points, longitudinal change was assessed using the difference between initial and final available eGFR values (ΔGFR).

### Statistical analysis

All statistical analyses were performed using the IBM SPSS software package, version 22.0. Quantitative variables were tested for normality using the Kolmogorov–Smirnov test. Normally distributed quantitative variables were reported as means and standard deviations (SDs), whereas non-normally distributed quantitative variables were reported as medians and interquartile ranges (IQRs). Qualitative presented as numbers or percentages (%). Changes in the dependent variables, including eGFR_initial and eGFR_final, were analyzed using the Wilcoxon signed-rank test. Comparisons among more than two groups were performed using the Kruskal–Wallis test, followed by *post-hoc* Dunn–Bonferroni correction for multiple comparisons. Associations between normally distributed continuous variables were assessed using Pearson's correlation coefficient. Normally distributed continuous variables were compared between two independent groups using the independent samples *t*-test, with variance homogeneity assessed by Levene's test. Results were considered statistically significant when *p* < 0.05. Multivariable linear regression was used to identify independent predictors of ΔGFR. Regression assumptions—including linearity, normality of residuals, homoscedasticity, independence of errors, and multicollinearity—were assessed prior to model fitting. Etiologic categories were incorporated into the analysis using dummy coding, with the unknown etiology group used as the reference category**.** All clinically relevant variables were entered into the initial model, and backward stepwise elimination was applied to obtain the most parsimonious model. Regression coefficients with 95% confidence intervals were reported, and statistical significance was set at *p* < 0.05.

## Results

A total of 73 patients were initially included in the study. The mean age at diagnosis was 4.85 ± 4.83 years, with a median of 3.0 years (IQR: 7.9). The study population included a slightly higher proportion of male patients (52.1%), with 42.5% being asymptomatic at presentation. Hypercalciuria was the most common metabolic risk factor for nephrocalcinosis, followed by hypocitraturia. The demographic and clinical characteristics of the patients are summarized in Table 1.

**Table 1:** Patient characteristics of nephrocalcinosis.

Patients characteristics	Number (%)
Total	73 (100%)
Males	38 (52.1%)
Presenting complaints	
Asymptomatic	31 (42.5%)
UTI-related symptoms	21 (28.8%)
Abdominal pain	9 (12.3%)
Stone passage	5 (6.8%)
Macroscopic hematuria	2 (2.7%)
Voiding dysfunction	1 (1.4%)
Failure to gain weight	1 (1.4%)
Vomiting	1 (1.4%)
Polyuria and polydipsia	1 (1.4%)
Muscle spasms	1 (1.4%)
Family history of urinary stone disease or nephrocalcinosis	28 (38.4%)
Consanguinity	21 (28.8%)
Other urinary tract anomalies	18 (24.6%)
Hydronephrosis	10 (13.6%)
Vesicoureteral reflux	3 (4.1%)
Ureteropelvic stenosis	2 (2.7%)
Kidney cyst	2 (2.7%)
Unilateral atrophic kidney	1 (1.4%)
UTI	31 (42.5%)
Recurrent UTIs	8 (11.0%)
Risk factors of nephrocalcinosis	
Hypercalciuria	40 (54.8%)
Hypocitraturia	21 (28.8%)
Hyperoxaluria	11 (15.1%)
Hyperuricosuria	7 (9.6%)
Cystinuria	1 (1.4%)

UTI, urinary tract infection.

The etiologic spectrum of nephrocalcinosis was heterogeneous, the most common underlying etiologies were hereditary/genetic and unknown causes (Table 2).

**Table 2:** Distribution of underlying etiologies and associated conditions in children with nephrocalcinosis (*n* = 73).

Etiology	Patients *n* (%)
Hereditary/Genetic	25 (34.2%)
dRTA	9 (12.3%)
FHHNC	7 (9.6%)
Bartter Syndrome	3 (4.1%)
Primary hyperoxaluria type 1	1 (1.4%)
Cystinuria	1 (1.4%)
Otoimmune polyglandular syndrome type 1	1 (1.4%)
Hypophosphatemic rickets	1 (1.4%)
Donohue syndrome	1 (1.4%)
Joubert syndrome	1 (1.4%)
Metabolic/Endocrine	16 (21.9%)
Idiopathic hypercalciuria	13 (17.8%)
Hypoparathyroidism	1 (1.4%)
Hyperuricemia	1 (1.4%)
Hypercalcemia due to fat necrosis	1 (1.4%)
Systemic/Syndromic	6 (8.2%)
Down syndrome	2 (2.7%)
Glycogen storage disease type 1b	1 (1.4%)
Epilepsy	1 (1.4%)
Prematurity	2 (2.7%)
Unknown	26 (35.6%)
Total	73 (100%)

dRTA, distal renal tubular acidosis; FHHNC, familial hypomagnesemia with hypercalciuria and nephrocalcinosis.

Of the initial cohort, follow-up data were available for 43 patients (58.9%), who were included in the longitudinal analyses, with a mean follow-up duration of 73.6 ± 58.2 months and a median of 52.0 months (IQR: 74.1). Mean eGFR values decreased significantly from initial to the final measurement (Table 3), as demonstrated by a Wilcoxon signed-rank test, *p* = 0.006.

**Table 3:** Comparison of eGFR values between initial and final measurements.

Variable	eGFR­_initial mean ± SD median (IQR)	eGFR_final mean ± SD median (IQR)	Difference (*Δ*GFR) mean ± SD median (IQR)	*p* value
eGFR (mL/min/1.73 m^2^)	117.9 ± 53.5 102.2 (53.7)	104.6 ± 58.3 95.9 (54.7)	13.3 ± 33.0 14.3 (42.3)	**0.006** *

eGFR, estimated glomerular filtration rate.

Bold values indicate statistical significance (*p* < 0.05).

* Wilcoxon signed-rank test.

**Figure 1:**
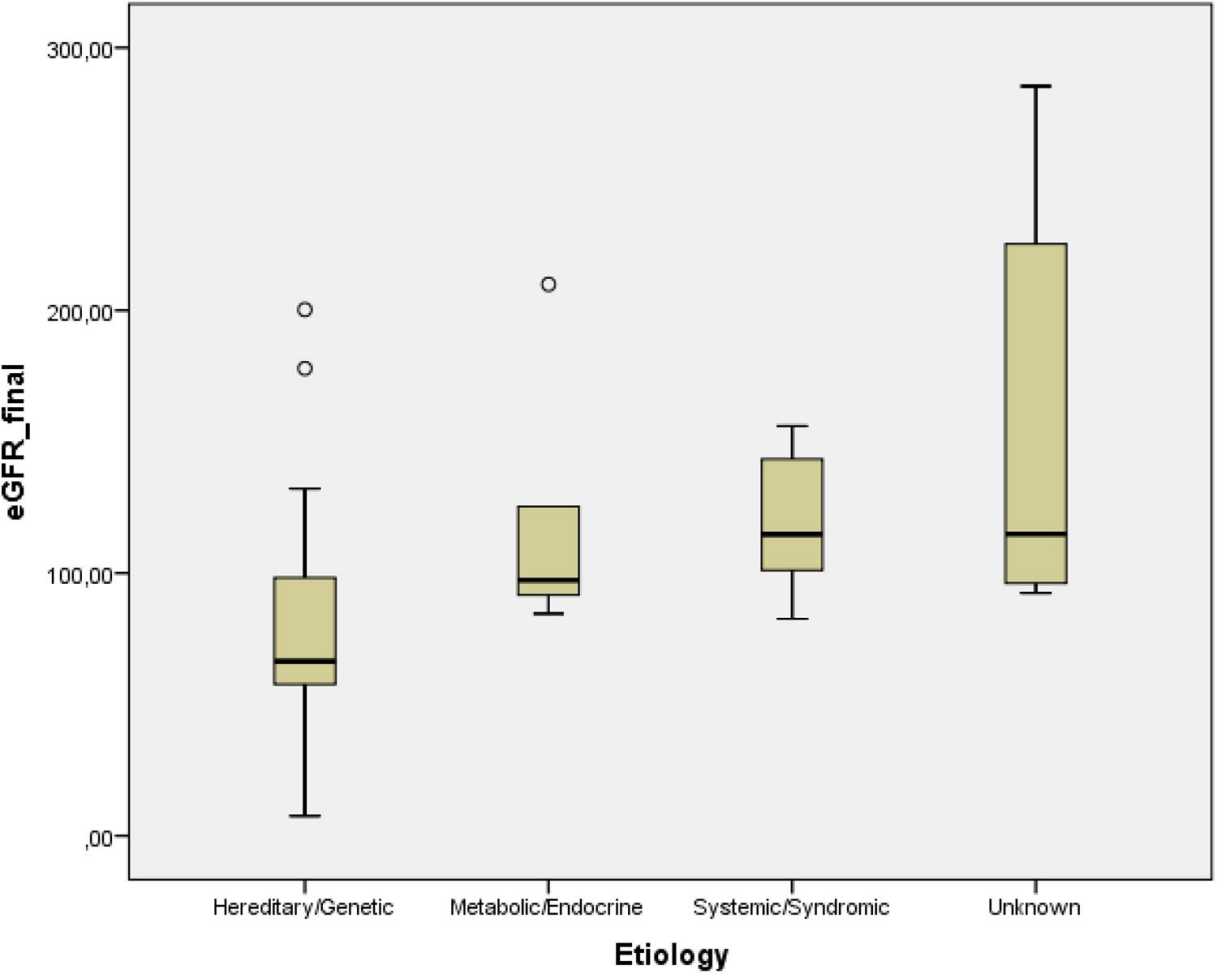
eGFR_final values according to etiology groups. *Post-hoc* Dunn–Bonferroni analysis showed significantly lower eGFR_final values in the hereditary/genetic group compared with the unknown etiology group (adjusted *p* = 0.015).

Comparisons of kidney function parameters according to etiology groups were subsequently performed to evaluate the impact of underlying conditions on eGFR­_initial, eGFR_final, and *Δ*GFR. eGFR­_initial and eGFR_final values differed significantly across etiology groups (Kruskal–Wallis test, *p* < 0.05 for both). *Post-hoc* Dunn–Bonferroni analysis revealed that patients in the hereditary/genetic etiology group had significantly lower eGFR_final values compared with those in the unknown etiology group (adjusted *p* = 0.015) (Figure 1). No other pairwise comparisons reached statistical significance. ΔGFR did not differ significantly among etiology groups (Table 4).

**Table 4:** Descriptive statistics of eGFR parameters according to etiology groups.

Etiology groups	Hereditary/Genetic (n: 23) mean ± SD median (IQR)	Metabolic/Endocrine (n: 6) mean ± SD median (IQR)	Systemic/Syndromic (n: 6) mean ± SD median (IQR)	Unknown (n: 8) mean ± SD median (IQR)	Total (n: 43) mean ± SD median (IQR)	*p* value
eGFR_initial (mL/min/1.73 m^2^)	99.1 ± 43.8 89.4 (47.2)	139.4 ± 64.2 126.5 (100.9)	126.0 ± 52.1 112.7 (62.5)	149.6 ± 59.1 130.9 (113.6)	117.9 ± 53.5 102.2 (53.7)	**0** **.** **050** *
eGFR_final (mL/min/1.73 m^2^)	79.4 ± 45.4 66.4 (42.4)	117.7 ± 47.3 97.3 (56.6)	118.8 ± 27.0 114.7 (50.1)	156.3 ± 79.9 114.9 (151.2)	104.6 ± 58.3 95.9 (54.7)	**0**.004*
*Δ*GFR (mL/min/1.73 m^2^)	19.7 ± 23.9 20.6 (32.5)	21.7 ± 34.6 15.9 (63.9)	7.2 ± 36.2 4.6 (44.9)	−6.7 ± 47.6 –0.1 (81.2)	13.3 ± 33.0 14.3 (42.3)	0.225**

Bold values indicate statistical significance (*p* < 0.05).

* Kruskal–Wallis test.

** One-way ANOVA test.

eGFR, estimated glomerular filtration rate.

A positive correlation was observed between ΔGFR and age at diagnosis (*r* = 0.313, *p* = 0.041), indicating that children diagnosed at an older age experienced a greater decline in kidney function. No significant correlation was observed between ΔGFR and follow-up duration.

ΔGFR did not differ significantly according to sex or the presence of metabolic risk factors, including hypercalciuria, hyperoxaluria, hypocitraturia, hyperuricosuria, and cystinuria (all *p* > 0.05). Similarly, no significant differences in *Δ*GFR were observed between patients with and without a history of urinary tract infection, recurrent urinary tract infections, or kidney anomalies.

Multivariable linear regression analysis was subsequently performed to identify independent predictors of ΔGFR. In the final multivariable model, ΔGFR was independently associated with history of UTI, systemic/syndromic etiology, and age at diagnosis. History of UTI (*β* = 23.0, 95% CI: 4.2–41.8, *p* = 0.018) and systemic/syndromic etiologies (*β* = 25.7, 95% CI: 3.3–48.2, *p* = 0.026) were associated with greater GFR decline, whereas age at diagnosis showed a positive linear relationship with ΔGFR (*β* = 2.03, 95% CI: 0.30–3.76, *p* = 0.022). Kidney anomaly demonstrated a borderline association (*β* = –19.6, 95% CI: −39.2 to 0.01, *p* = 0.050). The final model explained 26.7% of the variance in ΔGFR (adjusted *R*^2^ = 0.267) (Table 5).

**Table 5:** Multivariable linear regression model for predictors of ΔGFR (backward selection final model).

Predictor	*β* Coefficient	Std. Error	95% CI (Lower–Upper)	*p* value
History of UTI	23.016	9.283	4.223–41.809	**0** **.** **018**
Kidney anomaly	–19.602	9.688	–39.214 to –0.011	0.050
Age at diagnosis (years)	2.029	0.853	0.303–3.756	**0**.**022**
Systemic/Syndromic etiology^a^	25.739	11.104	3.260–48.218	**0**.**026**
Intercept	–19.784	11.472	–43.009 to –3.441	0.093

Model fit statistics: *R* = 0.581, *R*^2^ = 0.337, adjusted *R*^2^ = 0.267, standard error of the estimate = 28.26, Durbin–Watson = 1.97.

UTI, urinary tract infection.

Bold values indicate statistical significance (*p* < 0.05).

^a^ Systemic/syndromic etiology coded as 1 for systemic/syndromic causes and 0 for all other etiologic groups.

## Discussion

In the present study, we observed a significant decline in kidney function over time in a pediatric cohort with nephrocalcinosis, as reflected by lower final eGFR values compared with baseline after a median follow-up of 52 months. These observations indicate that nephrocalcinosis in childhood may not invariably follow a benign course, particularly during long-term follow-up. Consistent with our observations, Mantan et al. (9) observed a decline in GFR among children with nephrocalcinosis during follow-up, with an approximate 15% reduction after a median follow-up of 35 months, highlighting the potential for progressive kidney impairment in this population. Progressive kidney failure has been most consistently reported in a limited number of disorders associated with nephrocalcinosis, including primary hyperoxaluria, familial hypomagnesemia with hypercalciuria and nephrocalcinosis, Dent disease, and Lowe syndrome (5). In particular, nephrocalcinosis in primary hyperoxaluria has been reported to be associated with progression to end-stage kidney disease, with reported hazard ratios of 2.1 (95% CI 1.2–3.6) and 1.7 (95% CI 1.0–3.0) after adjustment in previous studies (10). In contrast, more favorable outcomes have also been reported; for example, a Korean study including 464 children with nephrocalcinosis (preterm: full-term ratio of approximately 3:1) documented resolution of nephrocalcinosis in nearly 62% of patients during follow-up (11). Taken together, although studies addressing the overall prognosis of nephrocalcinosis remain limited, available evidence indicates that kidney outcomes may vary substantially and are likely influenced by the underlying etiological factors.

Importantly, we found that eGFR­_final values differed significantly across etiological groups. Children with hereditary or genetic causes of nephrocalcinosis exhibited significantly lower final eGFR values compared with those with unknown etiology, supporting the notion that underlying disease mechanisms may play a more critical role in determining kidney outcome than nephrocalcinosis itself.

Although the magnitude of eGFR decline (ΔGFR) did not differ significantly among etiological groups in unadjusted analyses, this lack of difference may reflect heterogeneity in disease trajectories as well as the limited sample size within individual subgroups. Notably, when clinical and etiological variables were considered simultaneously, multivariable regression analysis identified systemic or syndromic etiology as an independent predictor of greater kidney function decline. Many systemic disorders, including genetic syndromes and storage diseases, are associated with an increased risk of kidney function decline through disease-specific mechanisms. For example, children with Down syndrome have a reduced kidney functional reserve and lower kidney function compared with healthy controls (12), while glycogen storage disease is characterized by kidney glycogen and lipid accumulation, leading to hyperfiltration, activation of the renin–angiotensin system, and podocyte injury (13). In addition, preterm birth has been linked to an increased risk of chronic kidney disease due to reduced nephron endowment at birth (14). Taken together, these findings suggest that in patients with systemic or syndromic conditions, kidney function decline may primarily reflect underlying disease-related kidney vulnerability, with nephrocalcinosis representing a concomitant rather than an independent contributor to progressive kidney damage. Our findings suggest the potential value of an individualized and multidisciplinary approach in the management of nephrocalcinosis in children with complex systemic diseases.

Additional **s**everal factors that independently contributed to a greater reduction in GFR. Our findings also demonstrate that a history of urinary tract infection, and older age at diagnosis are significant independent predictors of *Δ*GFR, while kidney anomalies show a borderline association. These findings underscore the importance of both underlying etiology and clinical course in determining long-term kidney outcomes in pediatric nephrocalcinosis.

In our cohort, urinary tract infections were common, with 42.5% of patients experiencing at least one UTI and 11.0% having recurrent infections. Previous studies have reported that 34%–40% of patients with nephrocalcinosis experience concomitant UTIs, which is comparable to the rate observed in our cohort (15, 16). In nephrocalcinosis, increased susceptibility to UTI has been partly attributed to hypercalciuria, as excess urinary calcium may enhance bacterial adherence to the urothelium, thereby increasing infection risk (17). This mechanism may contribute to the high prevalence of UTIs observed in children with nephrocalcinosis. It is well recognized that urinary tract infections can contribute to progressive kidney damage through inflammatory and fibrotic pathways (18). In our study, a history of UTI was identified as an independent factor associated with eGFR decline in multivariable analysis, suggesting that even a single episode may contribute to accelerated kidney injury in vulnerable kidneys, such as those already affected by nephrocalcinosis. Our results suggest that children with nephrocalcinosis and a history of UTI may constitute a higher-risk subgroup requiring closer surveillance and targeted infection prevention strategies. Given the observational design and the limited size of some etiological subgroups, the identified associations should be considered exploratory and interpreted with caution.

Age at diagnosis was included in multivariable analyses to account for potential age-related differences in baseline eGFR. In present study, age at diagnosis emerged as an independent factor associated with eGFR decline, and a positive correlation was observed between age at diagnosis and *Δ*GFR. It is well established that early diagnosis, prompt referral, and appropriate interventions to slow chronic kidney disease progression are important components of strategies to improve patient outcomes (19). Our finding indicates that children diagnosed at an older age may experience a greater decline in kidney function over time, potentially reflecting delayed recognition of nephrocalcinosis or prolonged exposure to underlying pathogenic mechanisms prior to diagnosis, although further studies are required to clarify this relationship.

Hereditary tubulopathies have been reported as a leading cause of nephrocalcinosis in pediatric populations (4, 15, 16), and in our cohort, they constituted the most common identified etiology following cases with unknown causes. Interestingly, traditional metabolic risk factors such as hypercalciuria, hyperoxaluria, and hypocitraturia were not independently associated with longitudinal changes in kidney function in our cohort. The lack of an independent association in multivariable analysis suggests that, when broader etiological and clinical factors are taken into account, isolated metabolic abnormalities alone may not sufficiently predict kidney function decline in this population. Nevertheless, hypercalciuria remained the most frequently identified metabolic risk factor in our cohort, followed by hypocitraturia, underscoring its central role in the pathogenesis of nephrocalcinosis even if its impact on long-term kidney outcome appears limited in this context.

This study has several limitations. First, its retrospective design inherently limits causal inference. Second, referral bias is likely, as the study population consisted of children followed at a tertiary care center, potentially enriching the cohort for more complex or severe cases. Third, the relatively small number of patients with longitudinal follow-up may have reduced the power to detect differences in eGFR decline across etiological subgroups. Fourth, an underlying cause of nephrocalcinosis could not be identified in 35.6% of patients, partly reflecting the fact that genetic testing was not performed systematically in all cases. In addition, nephrocalcinosis was not classified by the extent or localization of calcium deposition on ultrasonography, and kidney size parameters were not consistently available, limiting structural–functional correlations. Regression to the mean cannot be fully excluded, and unmeasured factors (e.g., nutritional status, intercurrent illnesses, AKI episodes, or hospitalizations) may have influenced eGFR estimates. Finally, incomplete follow-up in some patients may have limited the assessment of long-term kidney outcomes and may have introduced attrition bias.

In conclusion, our findings suggest that pediatric nephrocalcinosis may be associated with a decline in kidney function during long-term follow-up, and that kidney outcomes may be influenced by underlying etiological and clinical factors. Given that few studies have addressed the long-term prognosis of pediatric nephrocalcinosis, our study provides additional longitudinal data suggesting a potential impact of systemic or syndromic conditions, UTIs, and age at diagnosis on kidney outcomes. These findings support the importance of early diagnosis, careful etiological evaluation, individualized risk stratification, and close follow-up, particularly in higher-risk subgroups. Importantly, they also highlight the need for further prospective studies to better characterize prognostic factors and optimize long-term care in children with nephrocalcinosis.

## Data Availability

The raw data supporting the conclusions of this article will be made available by the authors, without undue reservation.
